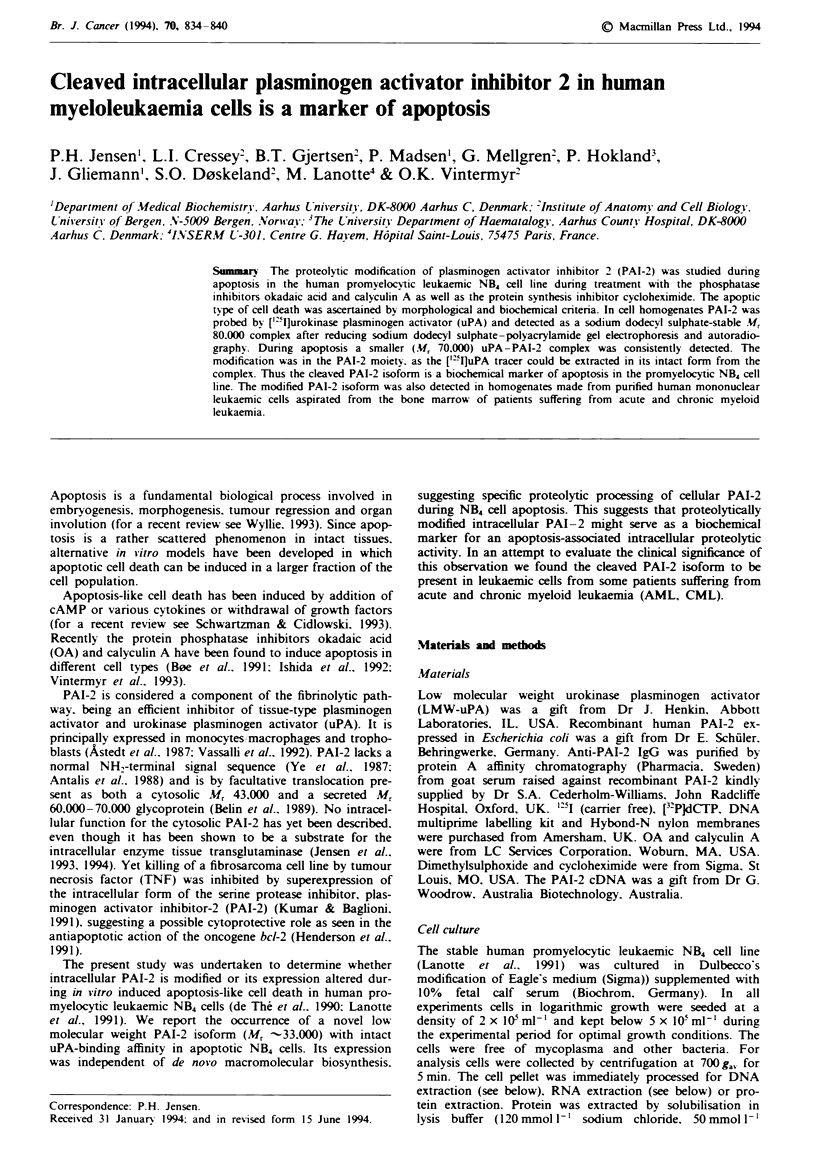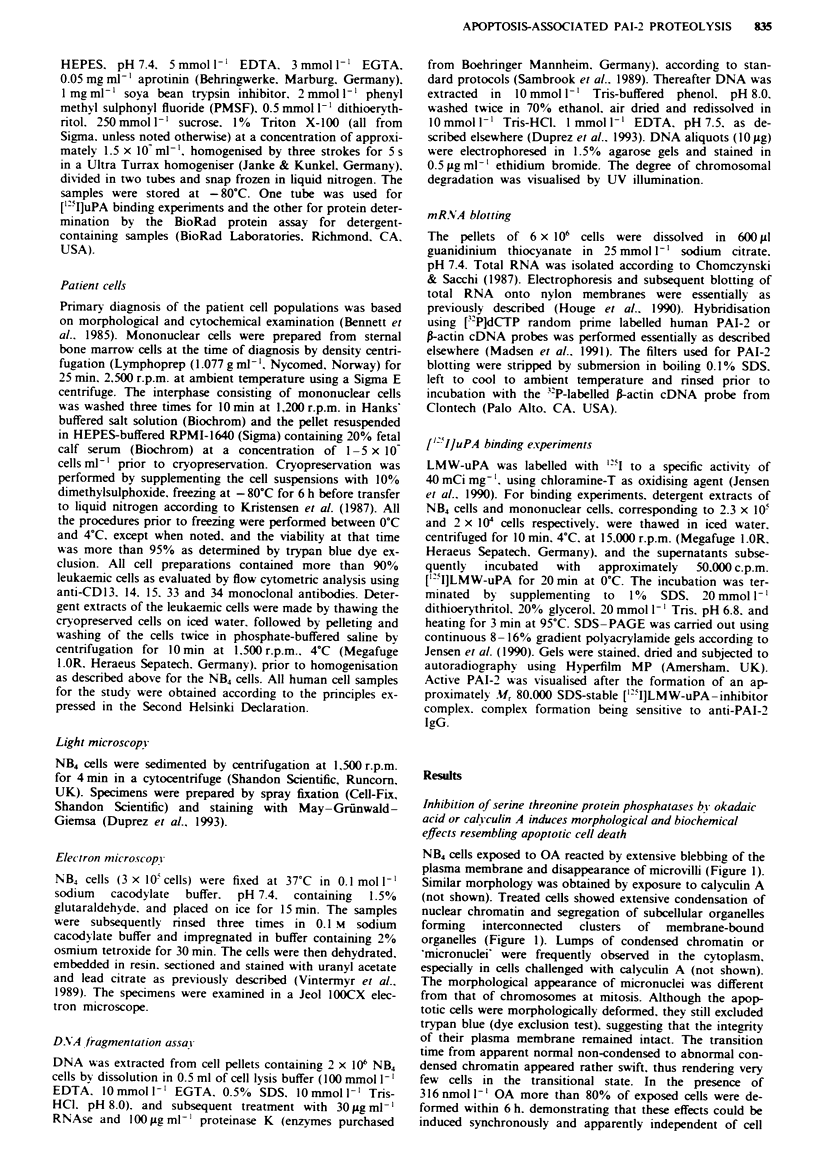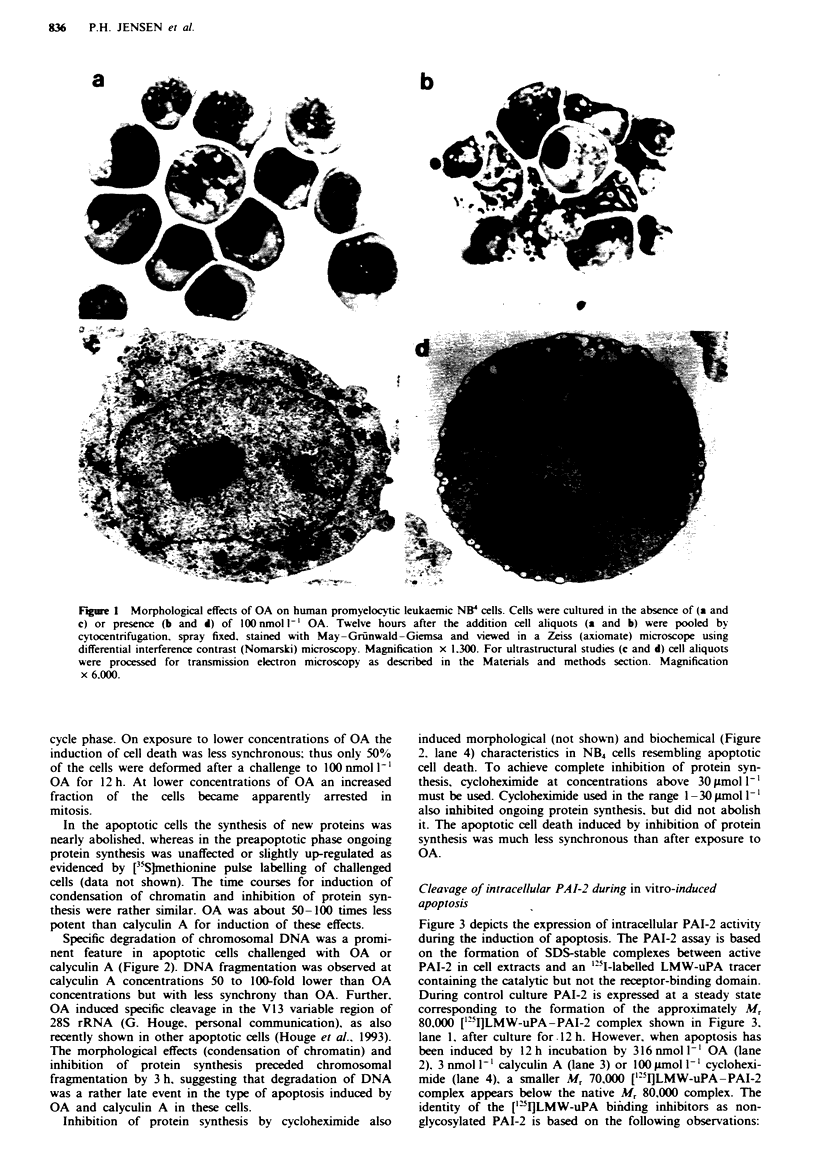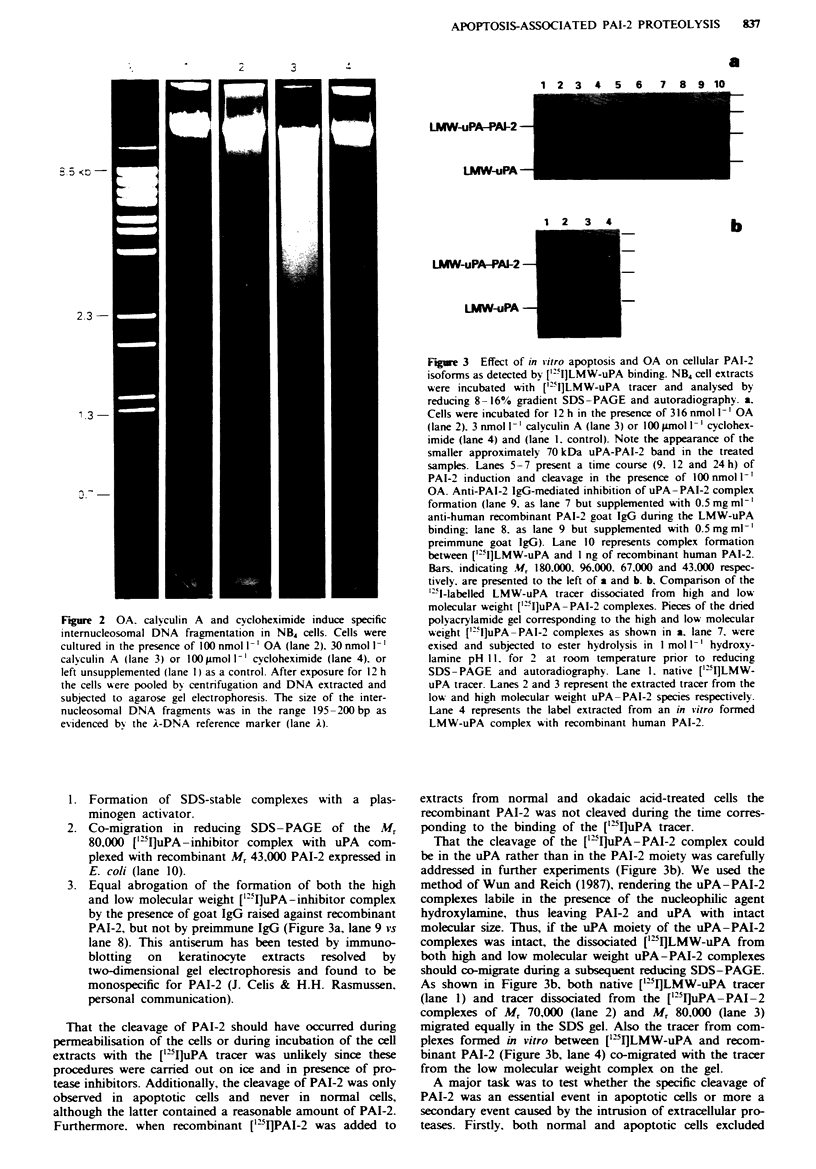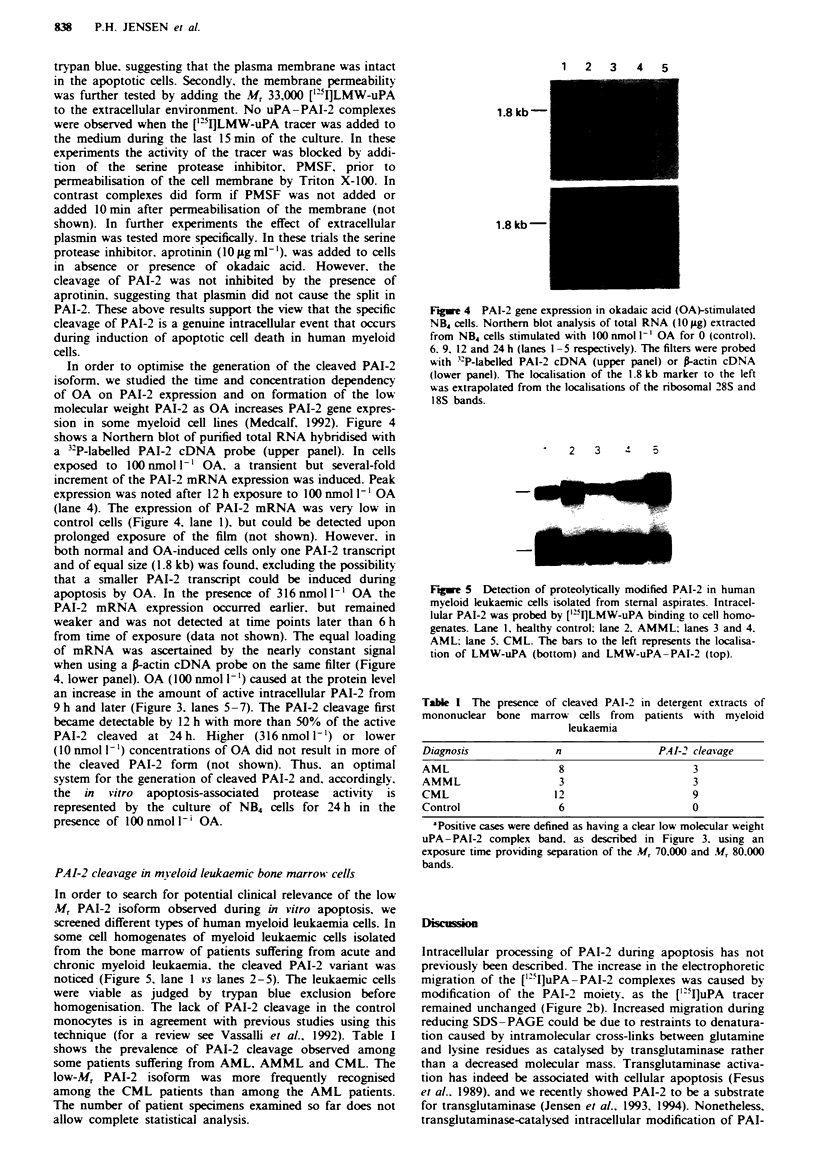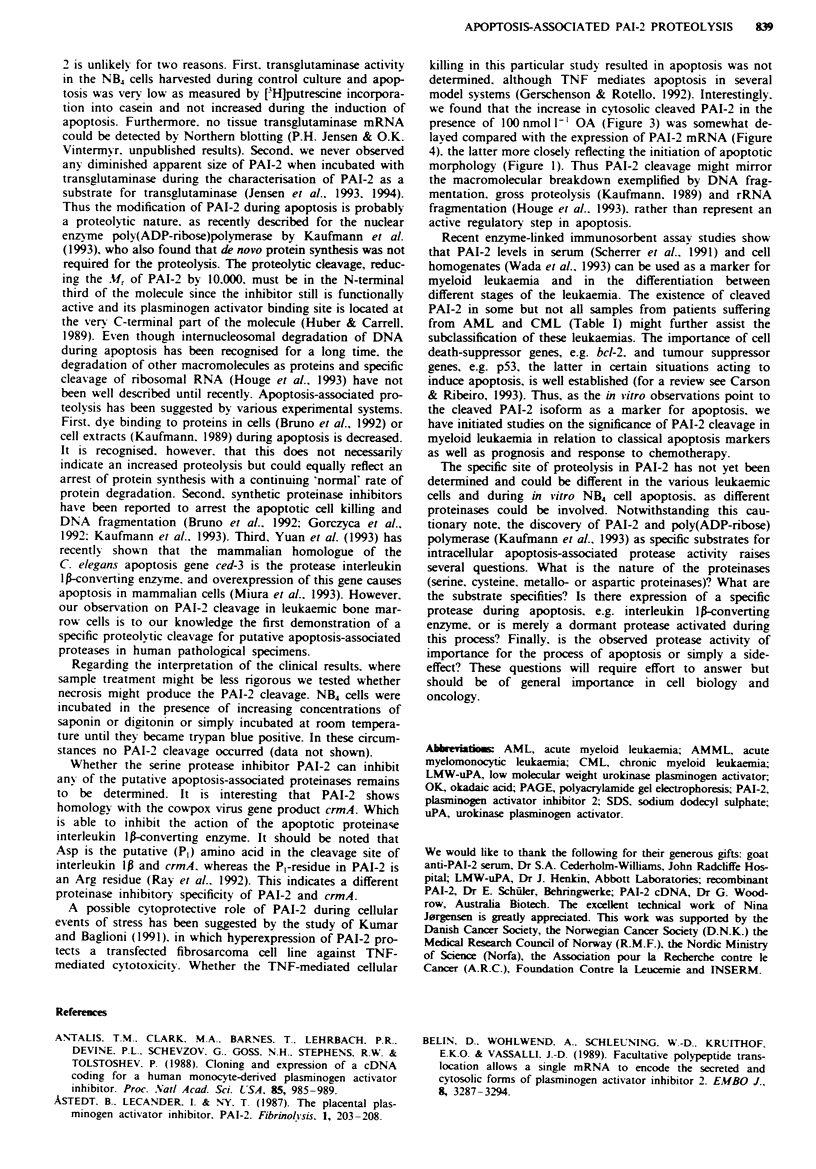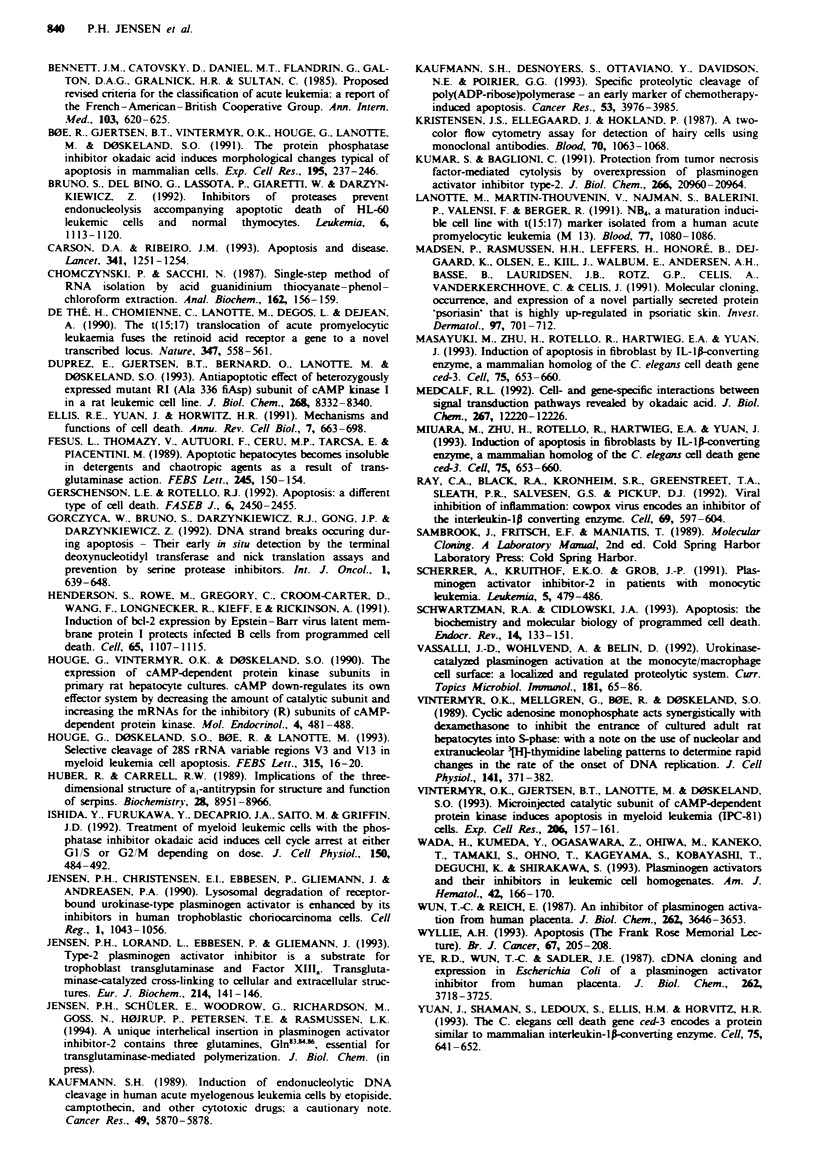# Cleaved intracellular plasminogen activator inhibitor 2 in human myeloleukaemia cells is a marker of apoptosis.

**DOI:** 10.1038/bjc.1994.407

**Published:** 1994-11

**Authors:** P. H. Jensen, L. I. Cressey, B. T. Gjertsen, P. Madsen, G. Mellgren, P. Hokland, J. Gliemann, S. O. Døskeland, M. Lanotte, O. K. Vintermyr

**Affiliations:** Department of Medical Biochemistry, Aarhus University, Denmark.

## Abstract

**Images:**


Br. J. Cancer (1994), 70, 834-840

© Macmillan Press Ltd., 1994

### Cleaved intracellular plasminogen activator inhibitor 2 in human myeloleukaemia cells is a marker of apoptosis

P.H. Jensen[1], L.I. Cressey[2], B.T. Gjertsen[2], P. Madsen[1], G. Mellgren[2], P. Hokland[3], J. Gliemann[1], S.O. Døskeland[2], M. Lanotte[4] & O.K. Vintermyr[2]

[1]*Department of Medical Biochemistry, Aarhus University, DK-8000 Aarhus C, Denmark;* [2]*Institute of Anatomy and Cell Biology, University of Bergen, N-5009 Bergen, Norway;* [3]*The University Department of Haematalogy, Aarhus County Hospital, DK-8000 Aarhus C, Denmark;* [4]*INSERM U-301, Centre G. Hayem, Hôpital Saint-Louis, 75475 Paris, France.*

**Summary** The proteolytic modification of plasminogen activator inhibitor 2 (PAI-2) was studied during apoptosis in the human promyelocytic leukaemic $NB_4$ cell line during treatment with the phosphatase inhibitors okadaic acid and calyculin A as well as the protein synthesis inhibitor cycloheximide. The apoptic type of cell death was ascertained by morphological and biochemical criteria. In cell homogenates PAI-2 was probed by [$^{125}$I]urokinase plasminogen activator (uPA) and detected as a sodium dodecyl sulphate-stable $M_r$ 80,000 complex after reducing sodium dodecyl sulphate-polyacrylamide gel electrophoresis and autoradiography. During apoptosis a smaller ($M_r$ 70,000) uPA-PAI-2 complex was consistently detected. The modification was in the PAI-2 moiety, as the [$^{125}$I]uPA tracer could be extracted in its intact form from the complex. Thus the cleaved PAI-2 isoform is a biochemical marker of apoptosis in the promyelocytic $NB_4$ cell line. The modified PAI-2 isoform was also detected in homogenates made from purified human mononuclear leukaemic cells aspirated from the bone marrow of patients suffering from acute and chronic myeloid leukaemia.

Apoptosis is a fundamental biological process involved in embryogenesis, morphogenesis, tumour regression and organ involution (for a recent review see Wyllie, 1993). Since apoptosis is a rather scattered phenomenon in intact tissues, alternative *in vitro* models have been developed in which apoptotic cell death can be induced in a larger fraction of the cell population.

Apoptosis-like cell death has been induced by addition of cAMP or various cytokines or withdrawal of growth factors (for a recent review see Schwartzman & Cidlowski, 1993). Recently the protein phosphatase inhibitors okadaic acid (OA) and calyculin A have been found to induce apoptosis in different cell types (Bøe *et al.*, 1991; Ishida *et al.*, 1992; Vintermyr *et al.*, 1993).

PAI-2 is considered a component of the fibrinolytic pathway, being an efficient inhibitor of tissue-type plasminogen activator and urokinase plasminogen activator (uPA). It is principally expressed in monocytes/macrophages and trophoblasts (Åstedt *et al.*, 1987; Vassalli *et al.*, 1992). PAI-2 lacks a normal $NH_2$-terminal signal sequence (Ye *et al.*, 1987; Antalis *et al.*, 1988) and is by facultative translocation present as both a cytosolic $M_r$ 43,000 and a secreted $M_r$ 60,000-70,000 glycoprotein (Belin *et al.*, 1989). No intracellular function for the cytosolic PAI-2 has yet been described, even though it has been shown to be a substrate for the intracellular enzyme tissue transglutaminase (Jensen *et al.*, 1993, 1994). Yet killing of a fibrosarcoma cell line by tumour necrosis factor (TNF) was inhibited by superexpression of the intracellular form of the serine protease inhibitor, plasminogen activator inhibitor-2 (PAI-2) (Kumar & Baglioni, 1991), suggesting a possible cytoprotective role as seen in the antiapoptotic action of the oncogene *bcl*-2 (Henderson *et al.*, 1991).

The present study was undertaken to determine whether intracellular PAI-2 is modified or its expression altered during *in vitro* induced apoptosis-like cell death in human promyelocytic leukaemic $NB_4$ cells (de Thé *et al.*, 1990; Lanotte *et al.*, 1991). We report the occurrence of a novel low molecular weight PAI-2 isoform ($M_r$ ~33,000) with intact uPA-binding affinity in apoptotic $NB_4$ cells. Its expression was independent of *de novo* macromolecular biosynthesis, suggesting specific proteolytic processing of cellular PAI-2 during $NB_4$ cell apoptosis. This suggests that proteolytically modified intracellular PAI-2 might serve as a biochemical marker for an apoptosis-associated intracellular proteolytic activity. In an attempt to evaluate the clinical significance of this observation we found the cleaved PAI-2 isoform to be present in leukaemic cells from some patients suffering from acute and chronic myeloid leukaemia (AML, CML).

Correspondence: P.H. Jensen.

Received 31 January 1994; and in revised form 15 June 1994.

### Materials and methods

### *Materials*

Low molecular weight urokinase plasminogen activator (LMW-uPA) was a gift from Dr J. Henkin, Abbott Laboratories, IL, USA. Recombinant human PAI-2 expressed in *Escherichia coli* was a gift from Dr E. Schüler, Behringwerke, Germany. Anti-PAI-2 IgG was purified by protein A affinity chromatography (Pharmacia, Sweden) from goat serum raised against recombinant PAI-2 kindly supplied by Dr S.A. Cederholm-Williams, John Radcliffe Hospital, Oxford, UK. $^{125}$I (carrier free), [$^{32}$P]dCTP, DNA multiprime labelling kit and Hybond-N nylon membranes were purchased from Amersham, UK. OA and calyculin A were from LC Services Corporation, Woburn, MA, USA. Dimethylsulphoxide and cycloheximide were from Sigma, St Louis, MO, USA. The PAI-2 cDNA was a gift from Dr G. Woodrow, Australia Biotechnology, Australia.

### *Cell culture*

The stable human promyelocytic leukaemic $NB_4$ cell line (Lanotte *et al.*, 1991) was cultured in Dulbecco's modification of Eagle's medium (Sigma)) supplemented with 10% fetal calf serum (Biochrom, Germany). In all experiments cells in logarithmic growth were seeded at a density of $2 \times 10^5$ ml$^{-1}$ and kept below $5 \times 10^5$ ml$^{-1}$ during the experimental period for optimal growth conditions. The cells were free of mycoplasma and other bacteria. For analysis cells were collected by centrifugation at 700 $g_{av}$ for 5 min. The cell pellet was immediately processed for DNA extraction (see below), RNA extraction (see below) or protein extraction. Protein was extracted by solubilisation in lysis buffer (120 mmol l$^{-1}$ sodium chloride, 50 mmol l$^{-1}$

HEPES, pH 7.4, 5 mmol $l^{-1}$ EDTA, 3 mmol $l^{-1}$ EGTA, 0.05 mg $ml^{-1}$ aprotinin (Behringwerke, Marburg, Germany), 1 mg $ml^{-1}$ soya bean trypsin inhibitor, 2 mmol $l^{-1}$ phenyl methyl sulphonyl fluoride (PMSF), 0.5 mmol $l^{-1}$ dithioerythritol, 250 mmol $l^{-1}$ sucrose, 1% Triton X-100 (all from Sigma, unless noted otherwise) at a concentration of approximately $1.5 \times 10^7$ $ml^{-1}$, homogenised by three strokes for 5 s in a Ultra Turrax homogeniser (Janke & Kunkel, Germany), divided in two tubes and snap frozen in liquid nitrogen. The samples were stored at −80°C. One tube was used for [$^{125}$I]uPA binding experiments and the other for protein determination by the BioRad protein assay for detergent-containing samples (BioRad Laboratories, Richmond, CA, USA).

*Patient cells*

Primary diagnosis of the patient cell populations was based on morphological and cytochemical examination (Bennett *et al.*, 1985). Mononuclear cells were prepared from sternal bone marrow cells at the time of diagnosis by density centrifugation (Lymphoprep (1.077 g $ml^{-1}$, Nycomed, Norway) for 25 min, 2,500 r.p.m. at ambient temperature using a Sigma E centrifuge. The interphase consisting of mononuclear cells was washed three times for 10 min at 1,200 r.p.m. in Hanks' buffered salt solution (Biochrom) and the pellet resuspended in HEPES-buffered RPMI-1640 (Sigma) containing 20% fetal calf serum (Biochrom) at a concentration of $1-5 \times 10^7$ cells $ml^{-1}$ prior to cryopreservation. Cryopreservation was performed by supplementing the cell suspensions with 10% dimethylsulphoxide, freezing at −80°C for 6 h before transfer to liquid nitrogen according to Kristensen *et al.* (1987). All the procedures prior to freezing were performed between 0°C and 4°C, except when noted, and the viability at that time was more than 95% as determined by trypan blue dye exclusion. All cell preparations contained more than 90% leukaemic cells as evaluated by flow cytometric analysis using anti-CD13, 14, 15, 33 and 34 monoclonal antibodies. Detergent extracts of the leukaemic cells were made by thawing the cryopreserved cells on iced water, followed by pelleting and washing of the cells twice in phosphate-buffered saline by centrifugation for 10 min at 1,500 r.p.m., 4°C (Megafuge 1.0R, Heraeus Sepatech, Germany), prior to homogenisation as described above for the $NB_4$ cells. All human cell samples for the study were obtained according to the principles expressed in the Second Helsinki Declaration.

*Light microscopy*

$NB_4$ cells were sedimented by centrifugation at 1,500 r.p.m. for 4 min in a cytocentrifuge (Shandon Scientific, Runcorn, UK). Specimens were prepared by spray fixation (Cell-Fix, Shandon Scientific) and staining with May–Grünwald–Giemsa (Duprez *et al.*, 1993).

*Electron microscopy*

$NB_4$ cells ($3 \times 10^5$ cells) were fixed at 37°C in 0.1 mol $l^{-1}$ sodium cacodylate buffer, pH 7.4, containing 1.5% glutaraldehyde, and placed on ice for 15 min. The samples were subsequently rinsed three times in 0.1 M sodium cacodylate buffer and impregnated in buffer containing 2% osmium tetroxide for 30 min. The cells were then dehydrated, embedded in resin, sectioned and stained with uranyl acetate and lead citrate as previously described (Vintermyr *et al.*, 1989). The specimens were examined in a Jeol 100CX electron microscope.

*DNA fragmentation assay*

DNA was extracted from cell pellets containing $2 \times 10^6$ $NB_4$ cells by dissolution in 0.5 ml of cell lysis buffer (100 mmol $l^{-1}$ EDTA, 10 mmol $l^{-1}$ EGTA, 0.5% SDS, 10 mmol $l^{-1}$ Tris-HCl, pH 8.0), and subsequent treatment with 30 $\mu$g $ml^{-1}$ RNAse and 100 $\mu$g $ml^{-1}$ proteinase K (enzymes purchased from Boehringer Mannheim, Germany), according to standard protocols (Sambrook *et al.*, 1989). Thereafter DNA was extracted in 10 mmol $l^{-1}$ Tris-buffered phenol, pH 8.0, washed twice in 70% ethanol, air dried and redissolved in 10 mmol $l^{-1}$ Tris-HCl, 1 mmol $l^{-1}$ EDTA, pH 7.5, as described elsewhere (Duprez *et al.*, 1993). DNA aliquots (10 $\mu$g) were electrophoresed in 1.5% agarose gels and stained in 0.5 $\mu$g $ml^{-1}$ ethidium bromide. The degree of chromosomal degradation was visualised by UV illumination.

*mRNA blotting*

The pellets of $6 \times 10^6$ cells were dissolved in 600 $\mu$l guanidinium thiocyanate in 25 mmol $l^{-1}$ sodium citrate, pH 7.4. Total RNA was isolated according to Chomczynski & Sacchi (1987). Electrophoresis and subsequent blotting of total RNA onto nylon membranes were essentially as previously described (Houge *et al.*, 1990). Hybridisation using [$^{32}$P]dCTP random prime labelled human PAI-2 or $\beta$-actin cDNA probes was performed essentially as described elsewhere (Madsen *et al.*, 1991). The filters used for PAI-2 blotting were stripped by submersion in boiling 0.1% SDS, left to cool to ambient temperature and rinsed prior to incubation with the $^{32}$P-labelled $\beta$-actin cDNA probe from Clontech (Palo Alto, CA, USA).

*[$^{125}$I]uPA binding experiments*

LMW-uPA was labelled with $^{125}$I to a specific activity of 40 mCi $mg^{-1}$, using chloramine-T as oxidising agent (Jensen *et al.*, 1990). For binding experiments, detergent extracts of $NB_4$ cells and mononuclear cells, corresponding to $2.3 \times 10^5$ and $2 \times 10^4$ cells respectively, were thawed in iced water, centrifuged for 10 min, 4°C, at 15,000 r.p.m. (Megafuge 1.0R, Heraeus Sepatech, Germany), and the supernatants subsequently incubated with approximately 50,000 c.p.m. [$^{125}$I]LMW-uPA for 20 min at 0°C. The incubation was terminated by supplementing to 1% SDS, 20 mmol $l^{-1}$ dithioerythritol, 20% glycerol, 20 mmol $l^{-1}$ Tris, pH 6.8, and heating for 3 min at 95°C. SDS–PAGE was carried out using continuous 8–16% gradient polyacrylamide gels according to Jensen *et al.* (1990). Gels were stained, dried and subjected to autoradiography using Hyperfilm MP (Amersham, UK). Active PAI-2 was visualised after the formation of an approximately $M_r$ 80,000 SDS-stable [$^{125}$I]LMW-uPA–inhibitor complex, complex formation being sensitive to anti-PAI-2 IgG.

### Results

*Inhibition of serine threonine protein phosphatases by okadaic acid or calyculin A induces morphological and biochemical effects resembling apoptotic cell death*

$NB_4$ cells exposed to OA reacted by extensive blebbing of the plasma membrane and disappearance of microvilli (Figure 1). Similar morphology was obtained by exposure to calyculin A (not shown). Treated cells showed extensive condensation of nuclear chromatin and segregation of subcellular organelles forming interconnected clusters of membrane-bound organelles (Figure 1). Lumps of condensed chromatin or 'micronuclei' were frequently observed in the cytoplasm, especially in cells challenged with calyculin A (not shown). The morphological appearance of micronuclei was different from that of chromosomes at mitosis. Although the apoptotic cells were morphologically deformed, they still excluded trypan blue (dye exclusion test), suggesting that the integrity of their plasma membrane remained intact. The transition time from apparent normal non-condensed to abnormal condensed chromatin appeared rather swift, thus rendering very few cells in the transitional state. In the presence of 316 nmol $l^{-1}$ OA more than 80% of exposed cells were deformed within 6 h, demonstrating that these effects could be induced synchronously and apparently independent of cell

**Figure 1** Morphological effects of OA on human promyelocytic leukaemic NB[4] cells. Cells were cultured in the absence of (**a** and **c**) or presence (**b** and **d**) of 100 nmol $l^{-1}$ OA. Twelve hours after the addition cell aliquots (**a** and **b**) were pooled by cytocentrifugation, spray fixed, stained with May-Grünwald-Giemsa and viewed in a Zeiss (axiomate) microscope using differential interference contrast (Nomarski) microscopy. Magnification × 1,300. For ultrastructural studies (**c** and **d**) cell aliquots were processed for transmission electron microscopy as described in the Materials and methods section. Magnification × 6,000.

cycle phase. On exposure to lower concentrations of OA the induction of cell death was less synchronous; thus only 50% of the cells were deformed after a challenge to 100 nmol $l^{-1}$ OA for 12 h. At lower concentrations of OA an increased fraction of the cells became apparently arrested in mitosis.

In the apoptotic cells the synthesis of new proteins was nearly abolished, whereas in the preapoptotic phase ongoing protein synthesis was unaffected or slightly up-regulated as evidenced by [$^{35}$S]methionine pulse labelling of challenged cells (data not shown). The time courses for induction of condensation of chromatin and inhibition of protein synthesis were rather similar. OA was about 50-100 times less potent than calyculin A for induction of these effects.

Specific degradation of chromosomal DNA was a prominent feature in apoptotic cells challenged with OA or calyculin A (Figure 2). DNA fragmentation was observed at calyculin A concentrations 50 to 100-fold lower than OA concentrations but with less synchrony than OA. Further, OA induced specific cleavage in the V13 variable region of 28S rRNA (G. Houge, personal communication), as also recently shown in other apoptotic cells (Houge *et al*., 1993). The morphological effects (condensation of chromatin) and inhibition of protein synthesis preceded chromosomal fragmentation by 3 h, suggesting that degradation of DNA was a rather late event in the type of apoptosis induced by OA and calyculin A in these cells.

Inhibition of protein synthesis by cycloheximide also induced morphological (not shown) and biochemical (Figure 2, lane 4) characteristics in $NB_4$ cells resembling apoptotic cell death. To achieve complete inhibition of protein synthesis, cycloheximide at concentrations above 30 μmol $l^{-1}$ must be used. Cycloheximide used in the range 1-30 μmol $l^{-1}$ also inhibited ongoing protein synthesis, but did not abolish it. The apoptotic cell death induced by inhibition of protein synthesis was much less synchronous than after exposure to OA.

### *Cleavage of intracellular PAI-2 during* in vitro-*induced apoptosis*

Figure 3 depicts the expression of intracellular PAI-2 activity during the induction of apoptosis. The PAI-2 assay is based on the formation of SDS-stable complexes between active PAI-2 in cell extracts and an $^{125}$I-labelled LMW-uPA tracer containing the catalytic but not the receptor-binding domain. During control culture PAI-2 is expressed at a steady state corresponding to the formation of the approximately $M_r$ 80,000 [$^{125}$I]LMW-uPA-PAI-2 complex shown in Figure 3, lane 1, after culture for 12 h. However, when apoptosis has been induced by 12 h incubation by 316 nmol $l^{-1}$ OA (lane 2), 3 nmol $l^{-1}$ calyculin A (lane 3) or 100 μmol $l^{-1}$ cycloheximide (lane 4), a smaller $M_r$ 70,000 [$^{125}$I]LMW-uPA-PAI-2 complex appears below the native $M_r$ 80,000 complex. The identity of the [$^{125}$I]LMW-uPA binding inhibitors as non-glycosylated PAI-2 is based on the following observations:

**Figure 2** OA, calyculin A and cycloheximide induce specific internucleosomal DNA fragmentation in $NB_4$ cells. Cells were cultured in the presence of 100 nmol $l^{-1}$ OA (lane 2), 30 nmol $l^{-1}$ calyculin A (lane 3) or 100 µmol $l^{-1}$ cycloheximide (lane 4), or left unsupplemented (lane 1) as a control. After exposure for 12 h the cells were pooled by centrifugation and DNA extracted and subjected to agarose gel electrophoresis. The size of the internucleosomal DNA fragments was in the range 195–200 bp as evidenced by the λ-DNA reference marker (lane λ).

**Figure 3** Effect of *in vitro* apoptosis and OA on cellular PAI-2 isoforms as detected by [$^{125}$I]LMW-uPA binding. $NB_4$ cell extracts were incubated with [$^{125}$I]LMW-uPA tracer and analysed by reducing 8–16% gradient SDS–PAGE and autoradiography. **a**, Cells were incubated for 12 h in the presence of 316 nmol $l^{-1}$ OA (lane 2), 3 nmol $l^{-1}$ calyculin A (lane 3) or 100 µmol $l^{-1}$ cycloheximide (lane 4) and (lane 1, control). Note the appearance of the smaller approximately 70 kDa uPA-PAI-2 band in the treated samples. Lanes 5–7 present a time course (9, 12 and 24 h) of PAI-2 induction and cleavage in the presence of 100 nmol $l^{-1}$ OA. Anti-PAI-2 IgG-mediated inhibition of uPA–PAI-2 complex formation (lane 9, as lane 7 but supplemented with 0.5 mg $ml^{-1}$ anti-human recombinant PAI-2 goat IgG during the LMW-uPA binding: lane 8, as lane 9 but supplemented with 0.5 mg $ml^{-1}$ preimmune goat IgG). Lane 10 represents complex formation between [$^{125}$I]LMW-uPA and 1 ng of recombinant human PAI-2. Bars, indicating $M_r$ 180,000, 96,000, 67,000 and 43,000 respectively, are presented to the left of **a** and **b**. **b**, Comparison of the $^{125}$I-labelled LMW-uPA tracer dissociated from high and low molecular weight [$^{125}$I]uPA–PAI-2 complexes. Pieces of the dried polyacrylamide gel corresponding to the high and low molecular weight [$^{125}$I]uPA–PAI-2 complexes as shown in **a**, lane 7, were exised and subjected to ester hydrolysis in 1 mol $l^{-1}$ hydroxylamine pH 11, for 2 at room temperature prior to reducing SDS–PAGE and autoradiography. Lane 1, native [$^{125}$I]LMW-uPA tracer. Lanes 2 and 3 represent the extracted tracer from the low and high molecular weight uPA–PAI-2 species respectively. Lane 4 represents the label extracted from an *in vitro* formed LMW-uPA complex with recombinant human PAI-2.

1. Formation of SDS-stable complexes with a plasminogen activator.
2. Co-migration in reducing SDS–PAGE of the $M_r$ 80,000 [$^{125}$I]uPA–inhibitor complex with uPA complexed with recombinant $M_r$ 43,000 PAI-2 expressed in *E. coli* (lane 10).
3. Equal abrogation of the formation of both the high and low molecular weight [$^{125}$I]uPA–inhibitor complex by the presence of goat IgG raised against recombinant PAI-2, but not by preimmune IgG (Figure 3a, lane 9 *vs* lane 8). This antiserum has been tested by immunoblotting on keratinocyte extracts resolved by two-dimensional gel electrophoresis and found to be monospecific for PAI-2 (J. Celis & H.H. Rasmussen, personal communication).

That the cleavage of PAI-2 should have occurred during permeabilisation of the cells or during incubation of the cell extracts with the [$^{125}$I]uPA tracer was unlikely since these procedures were carried out on ice and in presence of protease inhibitors. Additionally, the cleavage of PAI-2 was only observed in apoptotic cells and never in normal cells, although the latter contained a reasonable amount of PAI-2. Furthermore, when recombinant [$^{125}$I]PAI-2 was added to extracts from normal and okadaic acid-treated cells the recombinant PAI-2 was not cleaved during the time corresponding to the binding of the [$^{125}$I]uPA tracer.

That the cleavage of the [$^{125}$I]uPA–PAI-2 complex could be in the uPA rather than in the PAI-2 moiety was carefully addressed in further experiments (Figure 3b). We used the method of Wun and Reich (1987), rendering the uPA–PAI-2 complexes labile in the presence of the nucleophilic agent hydroxylamine, thus leaving PAI-2 and uPA with intact molecular size. Thus, if the uPA moiety of the uPA–PAI-2 complexes was intact, the dissociated [$^{125}$I]LMW-uPA from both high and low molecular weight uPA–PAI-2 complexes should co-migrate during a subsequent reducing SDS–PAGE. As shown in Figure 3b, both native [$^{125}$I]LMW-uPA tracer (lane 1) and tracer dissociated from the [$^{125}$I]uPA–PAI-2 complexes of $M_r$ 70,000 (lane 2) and $M_r$ 80,000 (lane 3) migrated equally in the SDS gel. Also the tracer from complexes formed *in vitro* between [$^{125}$I]LMW-uPA and recombinant PAI-2 (Figure 3b, lane 4) co-migrated with the tracer from the low molecular weight complex on the gel.

A major task was to test whether the specific cleavage of PAI-2 was an essential event in apoptotic cells or more a secondary event caused by the intrusion of extracellular proteases. Firstly, both normal and apoptotic cells excluded trypan blue, suggesting that the plasma membrane was intact in the apoptotic cells. Secondly, the membrane permeability was further tested by adding the $M_r$ 33,000 [$^{125}$I]LMW-uPA to the extracellular environment. No uPA-PAI-2 complexes were observed when the [$^{125}$I]LMW-uPA tracer was added to the medium during the last 15 min of the culture. In these experiments the activity of the tracer was blocked by addition of the serine protease inhibitor, PMSF, prior to permeabilisation of the cell membrane by Triton X-100. In contrast complexes did form if PMSF was not added or added 10 min after permeabilisation of the membrane (not shown). In further experiments the effect of extracellular plasmin was tested more specifically. In these trials the serine protease inhibitor, aprotinin (10 $\mu$g ml$^{-1}$), was added to cells in absence or presence of okadaic acid. However, the cleavage of PAI-2 was not inhibited by the presence of aprotinin, suggesting that plasmin did not cause the split in PAI-2. These above results support the view that the specific cleavage of PAI-2 is a genuine intracellular event that occurs during induction of apoptotic cell death in human myeloid cells.

In order to optimise the generation of the cleaved PAI-2 isoform, we studied the time and concentration dependency of OA on PAI-2 expression and on formation of the low molecular weight PAI-2 as OA increases PAI-2 gene expression in some myeloid cell lines (Medcalf, 1992). Figure 4 shows a Northern blot of purified total RNA hybridised with a $^{32}$P-labelled PAI-2 cDNA probe (upper panel). In cells exposed to 100 nmol l$^{-1}$ OA, a transient but several-fold increment of the PAI-2 mRNA expression was induced. Peak expression was noted after 12 h exposure to 100 nmol l$^{-1}$ OA (lane 4). The expression of PAI-2 mRNA was very low in control cells (Figure 4, lane 1), but could be detected upon prolonged exposure of the film (not shown). However, in both normal and OA-induced cells only one PAI-2 transcript and of equal size (1.8 kb) was found, excluding the possibility that a smaller PAI-2 transcript could be induced during apoptosis by OA. In the presence of 316 nmol l$^{-1}$ OA the PAI-2 mRNA expression occurred earlier, but remained weaker and was not detected at time points later than 6 h from time of exposure (data not shown). The equal loading of mRNA was ascertained by the nearly constant signal when using a $\beta$-actin cDNA probe on the same filter (Figure 4, lower panel). OA (100 nmol l$^{-1}$) caused at the protein level an increase in the amount of active intracellular PAI-2 from 9 h and later (Figure 3, lanes 5-7). The PAI-2 cleavage first became detectable by 12 h with more than 50% of the active PAI-2 cleaved at 24 h. Higher (316 nmol l$^{-1}$) or lower (10 nmol l$^{-1}$) concentrations of OA did not result in more of the cleaved PAI-2 form (not shown). Thus, an optimal system for the generation of cleaved PAI-2 and, accordingly, the *in vitro* apoptosis-associated protease activity is represented by the culture of $NB_4$ cells for 24 h in the presence of 100 nmol l$^{-1}$ OA.

### *PAI-2 cleavage in myeloid leukaemic bone marrow cells*

In order to search for potential clinical relevance of the low $M_r$ PAI-2 isoform observed during *in vitro* apoptosis, we screened different types of human myeloid leukaemia cells. In some cell homogenates of myeloid leukaemic cells isolated from the bone marrow of patients suffering from acute and chronic myeloid leukaemia, the cleaved PAI-2 variant was noticed (Figure 5, lane 1 *vs* lanes 2-5). The leukaemic cells were viable as judged by trypan blue exclusion before homogenisation. The lack of PAI-2 cleavage in the control monocytes is in agreement with previous studies using this technique (for a review see Vassalli *et al.*, 1992). Table I shows the prevalence of PAI-2 cleavage observed among some patients suffering from AML, AMML and CML. The low-$M_r$ PAI-2 isoform was more frequently recognised among the CML patients than among the AML patients. The number of patient specimens examined so far does not allow complete statistical analysis.

**Figure 4** PAI-2 gene expression in okadaic acid (OA)-stimulated $NB_4$ cells. Northern blot analysis of total RNA (10 $\mu$g) extracted from $NB_4$ cells stimulated with 100 nmol l$^{-1}$ OA for 0 (control), 6, 9, 12 and 24 h (lanes 1-5 respectively). The filters were probed with $^{32}$P-labelled PAI-2 cDNA (upper panel) or $\beta$-actin cDNA (lower panel). The localisation of the 1.8 kb marker to the left was extrapolated from the localisations of the ribosomal 28S and 18S bands.

**Figure 5** Detection of proteolytically modified PAI-2 in human myeloid leukaemic cells isolated from sternal aspirates. Intracellular PAI-2 was probed by [$^{125}$I]LMW-uPA binding to cell homogenates. Lane 1, healthy control; lane 2, AMML; lanes 3 and 4, AML; lane 5, CML. The bars to the left represents the localisation of LMW-uPA (bottom) and LMW-uPA-PAI-2 (top).

**Table I** The presence of cleaved PAI-2 in detergent extracts of mononuclear bone marrow cells from patients with myeloid leukaemia

| *Diagnosis* | *n* | *PAI-2 cleavage* |
|---|---|---|
| AML | 8 | 3 |
| AMML | 3 | 3 |
| CML | 12 | 9 |
| Control | 6 | 0 |

[a]Positive cases were defined as having a clear low molecular weight uPA-PAI-2 complex band, as described in Figure 3, using an exposure time providing separation of the $M_r$ 70,000 and $M_r$ 80,000 bands.

### Discussion

Intracellular processing of PAI-2 during apoptosis has not previously been described. The increase in the electrophoretic migration of the [$^{125}$I]uPA-PAI-2 complexes was caused by modification of the PAI-2 moiety, as the [$^{125}$I]uPA tracer remained unchanged (Figure 2b). Increased migration during reducing SDS-PAGE could be due to restraints to denaturation caused by intramolecular cross-links between glutamine and lysine residues as catalysed by transglutaminase rather than a decreased molecular mass. Transglutaminase activation has indeed be associated with cellular apoptosis (Fesus *et al.*, 1989), and we recently showed PAI-2 to be a substrate for transglutaminase (Jensen *et al.*, 1993, 1994). Nonetheless, transglutaminase-catalysed intracellular modification of PAI-

2 is unlikely for two reasons. First, transglutaminase activity in the $NB_4$ cells harvested during control culture and apoptosis was very low as measured by [$^3$H]putrescine incorporation into casein and not increased during the induction of apoptosis. Furthermore, no tissue transglutaminase mRNA could be detected by Northern blotting (P.H. Jensen & O.K. Vintermyr, unpublished results). Second, we never observed any diminished apparent size of PAI-2 when incubated with transglutaminase during the characterisation of PAI-2 as a substrate for transglutaminase (Jensen *et al.*, 1993, 1994). Thus the modification of PAI-2 during apoptosis is probably a proteolytic nature, as recently described for the nuclear enzyme poly(ADP-ribose)polymerase by Kaufmann *et al.* (1993), who also found that *de novo* protein synthesis was not required for the proteolysis. The proteolytic cleavage, reducing the $M_r$ of PAI-2 by 10,000, must be in the N-terminal third of the molecule since the inhibitor still is functionally active and its plasminogen activator binding site is located at the very C-terminal part of the molecule (Huber & Carrell, 1989). Even though internucleosomal degradation of DNA during apoptosis has been recognised for a long time, the degradation of other macromolecules as proteins and specific cleavage of ribosomal RNA (Houge *et al.*, 1993) have not been well described until recently. Apoptosis-associated proteolysis has been suggested by various experimental systems. First, dye binding to proteins in cells (Bruno *et al.*, 1992) or cell extracts (Kaufmann, 1989) during apoptosis is decreased. It is recognised, however, that this does not necessarily indicate an increased proteolysis but could equally reflect an arrest of protein synthesis with a continuing 'normal' rate of protein degradation. Second, synthetic proteinase inhibitors have been reported to arrest the apoptotic cell killing and DNA fragmentation (Bruno *et al.*, 1992; Gorczyca *et al.*, 1992; Kaufmann *et al.*, 1993). Third, Yuan *et al.* (1993) has recently shown that the mammalian homologue of the *C. elegans* apoptosis gene *ced*-3 is the protease interleukin 1β-converting enzyme, and overexpression of this gene causes apoptosis in mammalian cells (Miura *et al.*, 1993). However, our observation on PAI-2 cleavage in leukaemic bone marrow cells is to our knowledge the first demonstration of a specific proteolytic cleavage for putative apoptosis-associated proteases in human pathological specimens.

Regarding the interpretation of the clinical results, where sample treatment might be less rigorous we tested whether necrosis might produce the PAI-2 cleavage. $NB_4$ cells were incubated in the presence of increasing concentrations of saponin or digitonin or simply incubated at room temperature until they became trypan blue positive. In these circumstances no PAI-2 cleavage occurred (data not shown).

Whether the serine protease inhibitor PAI-2 can inhibit any of the putative apoptosis-associated proteinases remains to be determined. It is interesting that PAI-2 shows homology with the cowpox virus gene product *crmA*. Which is able to inhibit the action of the apoptotic proteinase interleukin 1β-converting enzyme. It should be noted that Asp is the putative ($P_1$) amino acid in the cleavage site of interleukin 1β and *crmA*, whereas the $P_1$-residue in PAI-2 is an Arg residue (Ray *et al.*, 1992). This indicates a different proteinase inhibitory specificity of PAI-2 and *crmA*.

A possible cytoprotective role of PAI-2 during cellular events of stress has been suggested by the study of Kumar and Baglioni (1991), in which hyperexpression of PAI-2 protects a transfected fibrosarcoma cell line against TNF-mediated cytotoxicity. Whether the TNF-mediated cellular killing in this particular study resulted in apoptosis was not determined, although TNF mediates apoptosis in several model systems (Gerschenson & Rotello, 1992). Interestingly, we found that the increase in cytosolic cleaved PAI-2 in the presence of 100 nmol $l^{-1}$ OA (Figure 3) was somewhat delayed compared with the expression of PAI-2 mRNA (Figure 4), the latter more closely reflecting the initiation of apoptotic morphology (Figure 1). Thus PAI-2 cleavage might mirror the macromolecular breakdown exemplified by DNA fragmentation, gross proteolysis (Kaufmann, 1989) and rRNA fragmentation (Houge *et al.*, 1993), rather than represent an active regulatory step in apoptosis.

Recent enzyme-linked immunosorbent assay studies show that PAI-2 levels in serum (Scherrer *et al.*, 1991) and cell homogenates (Wada *et al.*, 1993) can be used as a marker for myeloid leukaemia and in the differentiation between different stages of the leukaemia. The existence of cleaved PAI-2 in some but not all samples from patients suffering from AML and CML (Table I) might further assist the subclassification of these leukaemias. The importance of cell death-suppressor genes, e.g. *bcl*-2, and tumour suppressor genes, e.g. p53, the latter in certain situations acting to induce apoptosis, is well established (for a review see Carson & Ribeiro, 1993). Thus, as the *in vitro* observations point to the cleaved PAI-2 isoform as a marker for apoptosis, we have initiated studies on the significance of PAI-2 cleavage in myeloid leukaemia in relation to classical apoptosis markers as well as prognosis and response to chemotherapy.

The specific site of proteolysis in PAI-2 has not yet been determined and could be different in the various leukaemic cells and during *in vitro* $NB_4$ cell apoptosis, as different proteinases could be involved. Notwithstanding this cautionary note, the discovery of PAI-2 and poly(ADP-ribose) polymerase (Kaufmann *et al.*, 1993) as specific substrates for intracellular apoptosis-associated protease activity raises several questions. What is the nature of the proteinases (serine, cysteine, metallo- or aspartic proteinases)? What are the substrate specifities? Is there expression of a specific protease during apoptosis, e.g. interleukin 1β-converting enzyme, or is merely a dormant protease activated during this process? Finally, is the observed protease activity of importance for the process of apoptosis or simply a side-effect? These questions will require effort to answer but should be of general importance in cell biology and oncology.

**Abbreviations:** AML, acute myeloid leukaemia; AMML, acute myelomonocytic leukaemia; CML, chronic myeloid leukaemia; LMW-uPA, low molecular weight urokinase plasminogen activator; OK, okadaic acid; PAGE, polyacrylamide gel electrophoresis; PAI-2, plasminogen activator inhibitor 2; SDS, sodium dodecyl sulphate; uPA, urokinase plasminogen activator.

We would like to thank the following for their generous gifts: goat anti-PAI-2 serum, Dr S.A. Cederholm-Williams, John Radcliffe Hospital; LMW-uPA, Dr J. Henkin, Abbott Laboratories; recombinant PAI-2, Dr E. Schüler, Behringwerke; PAI-2 cDNA, Dr G. Woodrow, Australia Biotech. The excellent technical work of Nina Jørgensen is greatly appreciated. This work was supported by the Danish Cancer Society, the Norwegian Cancer Society (D.N.K.) the Medical Research Council of Norway (R.M.F.), the Nordic Ministry of Science (Norfa), the Association pour la Recherche contre le Cancer (A.R.C.), Foundation Contre la Leucemie and INSERM.

### References

ANTALIS, T.M., CLARK, M.A., BARNES, T., LEHRBACH, P.R., DEVINE, P.L., SCHEVZOV, G., GOSS, N.H., STEPHENS, R.W. & TOLSTOSHEV, P. (1988). Cloning and expression of a cDNA coding for a human monocyte-derived plasminogen activator inhibitor. *Proc. Natl Acad. Sci. USA*, **85**, 985–989.

ÅSTEDT, B., LECANDER, I. & NY, T. (1987). The placental plasminogen activator inhibitor, PAI-2. *Fibrinolysis*, **1**, 203–208.

BELIN, D., WOHLWEND, A., SCHLEUNING, W.-D., KRUITHOF, E.K.O. & VASSALLI, J.-D. (1989). Facultative polypeptide translocation allows a single mRNA to encode the secreted and cytosolic forms of plasminogen activator inhibitor 2. *EMBO J.*, **8**, 3287–3294.

BENNETT, J.M., CATOVSKY, D., DANIEL, M.T., FLANDRIN, G., GALTON, D.A.G., GRALNICK, H.R. & SULTAN, C. (1985). Proposed revised criteria for the classification of acute leukemia: a report of the French-American-British Cooperative Group. *Ann. Intern. Med.*, **103**, 620-625.

BØE, R., GJERTSEN, B.T., VINTERMYR, O.K., HOUGE, G., LANOTTE, M. & DØSKELAND, S.O. (1991). The protein phosphatase inhibitor okadaic acid induces morphological changes typical of apoptosis in mammalian cells. *Exp. Cell Res.*, **195**, 237-246.

BRUNO, S., DEL BINO, G., LASSOTA, P., GIARETTI, W. & DARZYNKIEWICZ, Z. (1992). Inhibitors of proteases prevent endonucleolysis accompanying apoptotic death of HL-60 leukemic cells and normal thymocytes. *Leukemia*, **6**, 1113-1120.

CARSON, D.A. & RIBEIRO, J.M. (1993). Apoptosis and disease. *Lancet*, **341**, 1251-1254.

CHOMCZYNSKI, P. & SACCHI, N. (1987). Single-step method of RNA isolation by acid guanidinium thiocyanate-phenol-chloroform extraction. *Anal. Biochem.*, **162**, 156-159.

DE THÉ, H., CHOMIENNE, C., LANOTTE, M., DEGOS, L. & DEJEAN, A. (1990). The t(15;17) translocation of acute promyelocytic leukaemia fuses the retinoid acid receptor a gene to a novel transcribed locus. *Nature*, **347**, 558-561.

DUPREZ, E., GJERTSEN, B.T., BERNARD, O., LANOTTE, M. & DØSKELAND, S.O. (1993). Antiapoptotic effect of heterozygously expressed mutant RI (Ala 336 fiAsp) subunit of cAMP kinase I in a rat leukemic cell line. *J. Biol. Chem.*, **268**, 8332-8340.

ELLIS, R.E., YUAN, J. & HORWITZ, H.R. (1991). Mechanisms and functions of cell death. *Annu. Rev. Cell Biol.*, **7**, 663-698.

FESUS, L., THOMAZY, V., AUTUORI, F., CERU, M.P., TARCSA, E. & PIACENTINI, M. (1989). Apoptotic hepatocytes becomes insoluble in detergents and chaotropic agents as a result of transglutaminase action. *FEBS Lett.*, **245**, 150-154.

GERSCHENSON, L.E. & ROTELLO, R.J. (1992). Apoptosis: a different type of cell death. *FASEB J.*, **6**, 2450-2455.

GORCZYCA, W., BRUNO, S., DARZYNKIEWICZ, R.J., GONG, J.P. & DARZYNKIEWICZ, Z. (1992). DNA strand breaks occuring during apoptosis - Their early *in situ* detection by the terminal deoxynucleotidyl transferase and nick translation assays and prevention by serine protease inhibitors. *Int. J. Oncol.*, **1**, 639-648.

HENDERSON, S., ROWE, M., GREGORY, C., CROOM-CARTER, D., WANG, F., LONGNECKER, R., KIEFF, E & RICKINSON, A. (1991). Induction of bcl-2 expression by Epstein-Barr virus latent membrane protein I protects infected B cells from programmed cell death. *Cell*, **65**, 1107-1115.

HOUGE, G., VINTERMYR, O.K. & DØSKELAND, S.O. (1990). The expression of cAMP-dependent protein kinase subunits in primary rat hepatocyte cultures. cAMP down-regulates its own effector system by decreasing the amount of catalytic subunit and increasing the mRNAs for the inhibitory (R) subunits of cAMP-dependent protein kinase. *Mol. Endocrinol.*, **4**, 481-488.

HOUGE, G., DØSKELAND, S.O., BØE, R. & LANOTTE, M. (1993). Selective cleavage of 28S rRNA variable regions V3 and V13 in myeloid leukemia cell apoptosis. *FEBS Lett.*, **315**, 16-20.

HUBER, R. & CARRELL, R.W. (1989). Implications of the three-dimensional structure of $a_1$-antitrypsin for structure and function of serpins. *Biochemistry*, **28**, 8951-8966.

ISHIDA, Y., FURUKAWA, Y., DECAPRIO, J.A., SAITO, M. & GRIFFIN, J.D. (1992). Treatment of myeloid leukemic cells with the phosphatase inhibitor okadaic acid induces cell cycle arrest at either G1/S or G2/M depending on dose. *J. Cell Physiol.*, **150**, 484-492.

JENSEN, P.H., CHRISTENSEN, E.I., EBBESEN, P., GLIEMANN, J. & ANDREASEN, P.A. (1990). Lysosomal degradation of receptor-bound urokinase-type plasminogen activator is enhanced by its inhibitors in human trophoblastic choriocarcinoma cells. *Cell Reg.*, **1**, 1043-1056.

JENSEN, P.H., LORAND, L., EBBESEN, P. & GLIEMANN, J. (1993). Type-2 plasminogen activator inhibitor is a substrate for trophoblast transglutaminase and Factor $XIII_a$. Transglutaminase-catalyzed cross-linking to cellular and extracellular structures. *Eur. J. Biochem.*, **214**, 141-146.

JENSEN, P.H., SCHÜLER, E., WOODROW, G., RICHARDSON, M., GOSS, N., HØJRUP, P., PETERSEN, T.E. & RASMUSSEN, L.K. (1994). A unique interhelical insertion in plasminogen activator inhibitor-2 contains three glutamines, $Gln^{83,84,86}$, essential for transglutaminase-mediated polymerization. *J. Biol. Chem.* (in press).

KAUFMANN, S.H. (1989). Induction of endonucleolytic DNA cleavage in human acute myelogenous leukemia cells by etopiside, camptothecin, and other cytotoxic drugs; a cautionary note. *Cancer Res.*, **49**, 5870-5878.

KAUFMANN, S.H., DESNOYERS, S., OTTAVIANO, Y., DAVIDSON, N.E. & POIRIER, G.G. (1993). Specific proteolytic cleavage of poly(ADP-ribose)polymerase - an early marker of chemotherapy-induced apoptosis. *Cancer Res.*, **53**, 3976-3985.

KRISTENSEN, J.S., ELLEGAARD, J. & HOKLAND, P. (1987). A two-color flow cytometry assay for detection of hairy cells using monoclonal antibodies. *Blood*, **70**, 1063-1068.

KUMAR, S. & BAGLIONI, C. (1991). Protection from tumor necrosis factor-mediated cytolysis by overexpression of plasminogen activator inhibitor type-2. *J. Biol. Chem.*, **266**, 20960-20964.

LANOTTE, M., MARTIN-THOUVENIN, V., NAJMAN, S., BALERINI, P., VALENSI, F. & BERGER, R. (1991). $NB_4$, a maturation inducible cell line with t(15:17) marker isolated from a human acute promyelocytic leukemia (M 13). *Blood*, **77**, 1080-1086.

MADSEN, P., RASMUSSEN, H.H., LEFFERS, H., HONORÉ, B., DEJGAARD, K., OLSEN, E., KIIL, J., WALBUM, E., ANDERSEN, A.H., BASSE, B., LAURIDSEN, J.B., ROTZ, G.P., CELIS, A., VANDERKERCHHOVE, C. & CELIS, J. (1991). Molecular cloning, occurrence, and expression of a novel partially secreted protein 'psoriasin' that is highly up-regulated in psoriatic skin. *Invest. Dermatol.*, **97**, 701-712.

MASAYUKI, M., ZHU, H., ROTELLO, R., HARTWIEG, E.A. & YUAN, J. (1993). Induction of apoptosis in fibroblast by IL-1β-converting enzyme, a mammalian homolog of the *C. elegans* cell death gene *ced*-3. *Cell*, **75**, 653-660.

MEDCALF, R.L. (1992). Cell- and gene-specific interactions between signal transduction pathways revealed by okadaic acid. *J. Biol. Chem.*, **267**, 12220-12226.

MIUARA, M., ZHU, H., ROTELLO, R., HARTWIEG, E.A. & YUAN, J. (1993). Induction of apoptosis in fibroblasts by IL-1β-converting enzyme, a mammalian homolog of the *C. elegans* cell death gene *ced-3*. *Cell*, **75**, 653-660.

RAY, C.A., BLACK, R.A., KRONHEIM, S.R., GREENSTREET, T.A., SLEATH, P.R., SALVESEN, G.S. & PICKUP, D.J. (1992). Viral inhibition of inflammation: cowpox virus encodes an inhibitor of the interleukin-1β converting enzyme. *Cell*, **69**, 597-604.

SAMBROOK, J., FRITSCH, E.F. & MANIATIS, T. (1989). *Molecular Cloning. A Laboratory Manual*, 2nd ed. Cold Spring Harbor Laboratory Press: Cold Spring Harbor.

SCHERRER, A., KRUITHOF, E.K.O. & GROB, J.-P. (1991). Plasminogen activator inhibitor-2 in patients with monocytic leukemia. *Leukemia*, **5**, 479-486.

SCHWARTZMAN, R.A. & CIDLOWSKI, J.A. (1993). Apoptosis: the biochemistry and molecular biology of programmed cell death. *Endocr. Rev.*, **14**, 133-151.

VASSALLI, J.-D., WOHLVEND, A. & BELIN, D. (1992). Urokinase-catalyzed plasminogen activation at the monocyte/macrophage cell surface: a localized and regulated proteolytic system. *Curr. Topics Microbiol. Immunol.*, **181**, 65-86.

VINTERMYR, O.K., MELLGREN, G., BØE, R. & DØSKELAND, S.O. (1989). Cyclic adenosine monophosphate acts synergistically with dexamethasone to inhibit the entrance of cultured adult rat hepatocytes into S-phase: with a note on the use of nucleolar and extranucleolar $^3$[H]-thymidine labeling patterns to determine rapid changes in the rate of the onset of DNA replication. *J. Cell Physiol.*, **141**, 371-382.

VINTERMYR, O.K., GJERTSEN, B.T., LANOTTE, M. & DØSKELAND, S.O. (1993). Microinjected catalytic subunit of cAMP-dependent protein kinase induces apoptosis in myeloid leukemia (IPC-81) cells. *Exp. Cell Res.*, **206**, 157-161.

WADA, H., KUMEDA, Y., OGASAWARA, Z., OHIWA, M., KANEKO, T., TAMAKI, S., OHNO, T., KAGEYAMA, S., KOBAYASHI, T., DEGUCHI, K. & SHIRAKAWA, S. (1993). Plasminogen activators and their inhibitors in leukemic cell homogenates. *Am. J. Hematol.*, **42**, 166-170.

WUN, T.-C. & REICH, E. (1987). An inhibitor of plasminogen activation from human placenta. *J. Biol. Chem.*, **262**, 3646-3653.

WYLLIE, A.H. (1993). Apoptosis (The Frank Rose Memorial Lecture). *Br. J. Cancer*, **67**, 205-208.

YE, R.D., WUN, T.-C. & SADLER, J.E. (1987). cDNA cloning and expression in *Escherichia Coli* of a plasminogen activator inhibitor from human placenta. *J. Biol. Chem.*, **262**, 3718-3725.

YUAN, J., SHAMAN, S., LEDOUX, S., ELLIS, H.M. & HORVITZ, H.R. (1993). The C. elegans cell death gene *ced*-3 encodes a protein similar to mammalian interleukin-1β-converting enzyme. *Cell*, **75**, 641-652.